# Crystal structure, Hirshfeld surface and inter­molecular inter­action energy analysis of the halogen-bonded 1:1 cocrystal 1-bromo-3,5-di­nitro­benzene–*N*,*N*-di­methyl­pyridin-4-amine

**DOI:** 10.1107/S2056989025006735

**Published:** 2025-08-05

**Authors:** Eric Bosch

**Affiliations:** a Chemistry and Biochemistry Department Missouri State University,Springfield MO 65897 USA; University of Missouri-Columbia, USA

**Keywords:** crystal structure, *N*,*N*-di­methyl­pyridin-4-amine, halogen bond, cocrystal, 1-bromo-3,5-di­nitro­benzene, π-stacking, donor–acceptor complex

## Abstract

The structure of the 1:1 cocrystal formed between 1-bromo-3,5-di­nitro­benzene and *N*,*N*-di­methyl­pyridin-4-amine that features a C—Br⋯N halogen bond is reported. The cocrystal, C_6_H_3_BrN_2_O_4_·C_7_H_10_N_2_, crystalizes in the monoclinic space group *P*2_1_/*c* with *Z* = 4. Hirshfeld surface analysis and inter­molecular inter­action energies within the cocrystal structure are reported.

## Abstract 2

The structure and analysis of the 1:1 cocrystal formed between 1-bromo-3,5-di­nitro­benzene and *N*,*N*-di­methyl­pyridin-4-amine is reported. Hirshfeld surface analysis and inter­molecular inter­action energies within the cocrystal structure are reported.

## 1. Chemical context

Halogen bonding is widely recognized as a versatile tool in mol­ecular recognition, supra­molecular chemistry, and crystal engineering (Cavallo *et al.*, 2016 ▸; Costa, 2017 ▸). Fundamental studies comparing the variation in halogen-bond strength for a variety of C–*-X* halogen-bond donors established that the strength of the halogen bond decreaseds in the order I > Br > Cl. Furthermore, the addition of electron-withdrawing atoms and/or substituents near a bonded halogen increases the halogen-bond strength. While fluorinated iodo­benzenes are common halogen-bond donors (Prasang *et al.*, 2009 ▸), the strong effect of nitro substituents on halogen-bond strength has been demonstrated in several different systems (Goud *et al.*, 2016 ▸; Nwachukwu *et al.*, 2018 ▸; Panikkattu *et al.*, 2022 ▸). Lewis bases, in particular pyridines, have been widely used as halogen-bond acceptors. In terms of simple pyridines, *N*,*N*-di­methyl­pyridin-4-amine as a strong organic base is a strong halogen-bond acceptor. Previously we published the structure of the cocrystal 1-iodo-3,5-di­nitro­benzene–*N*,*N*-di­methyl­pyridin-4-amine (Nwachukwu *et al.*, 2018 ▸) and here we report the 1:1 cocrystal 1-bromo-3,5-di­nitro­benzene–*N*,*N*-di­methyl­pyridin-4-amine (**BDNB·DMAP**). The Hirshfeld surface was analyzed and inter­molecular inter­action energies calculated.
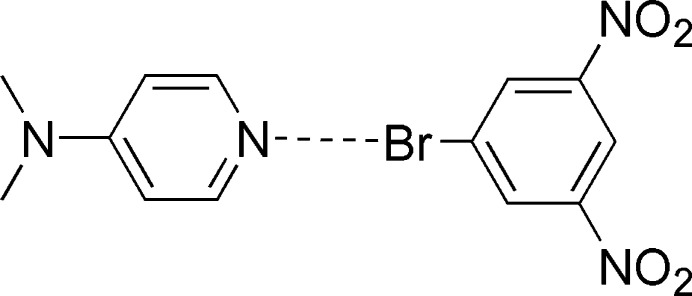


## 2. Structural commentary

**Figure 1:**
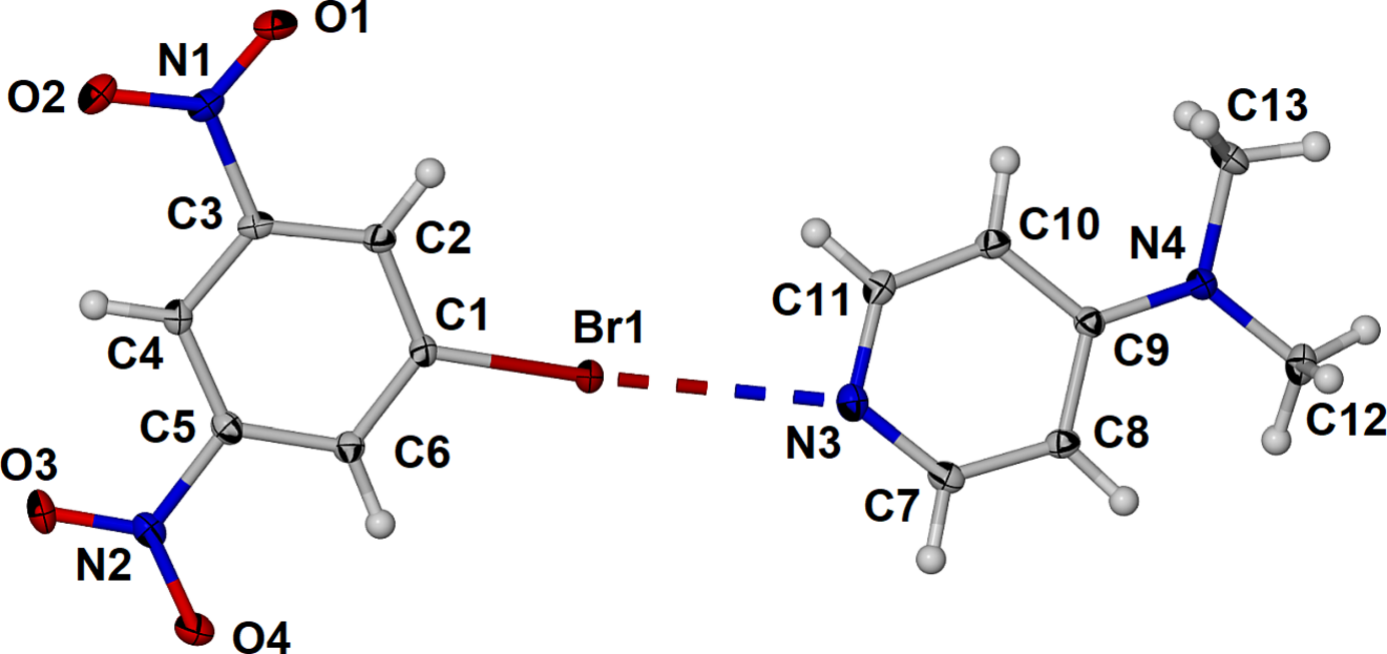
Asymmetric unit of the cocrystal **BDNB·DMAP** with displacement ellipsoids drawn at the 50% level and the halogen bond shown as a dashed line.

The asymmetric unit of the **BDNB·DMAP** cocrystal contains one mol­ecule of each component with a Br⋯N halogen bond as shown in Fig. 1 ▸. The pyridine and benzene mol­ecules are almost coplanar with twist and fold angles of 17.23 (10) and 6.53 (10)°, respectively. The Br⋯N separation is 3.004 (2) Å at 86% of the sum of the van der Waals radii (Bondi, 1964 ▸) while the C1—Br⋯N3 angle is essentially linear at 175.38 (10)°. The twist of the **DMAP** relative to **BDNB** is manifested in the N4—N3⋯Br1 angle of 155.56 (8) °.

The nitro moieties are essentially coplanar with the benzene core of **BDNB** with deviations above the benzene plane, C1–C6, of 0.012 (4) and 0.017 (4) Å for N1 and N2, respectively. The oxygen atoms O1 and O4 are 0.091 (5) and 0.056 (5) Å above the C1–C6 plane while O2 and O3 are 0.063 (5) and 0.26 (4) Å below the plane. The bromine atom is 0.106 (4) Å below the plane defined by the benzene ring. The C—C bond distances around the benzene ring are similar ranging from 1.376 (4) Å for C3—C4 to 1.387 (4) Å for C1—C6. The structure of **BDNB** alone was reported at 250 K (refcode: WUNNOL; Voutier *et al.*, 2020 ▸) with C—C bond distances ranging from 1.372 (16) to 1.40 (2) Å. The N—O distances in the **BDNB·DMAP** cocrystal range from 1.218 (3) to 1.230 (3) Å while those in **BDNB** alone are similar ranging from 1.207 (16) to 1.230 (15) Å. The di­methyl­amino group is also essentially coplanar with the plane defined by the pyridine ring, N3–C11, with the methyl carbon C12 0.151 (5) Å below and methyl carbon C13 0.070 (5) Å above the pyridyl plane. The C—C and C—N bond distances within the pyridyl ring of **DMAP** are in good agreement with those reported in the structure of **DMAP** alone at 123 K (refcode: BUKJOG16; Nieger, 2022 ▸) consistent with distortion due to the contribution of the polarized resonance form.

## 3. Supra­molecular features

**Figure 2:**
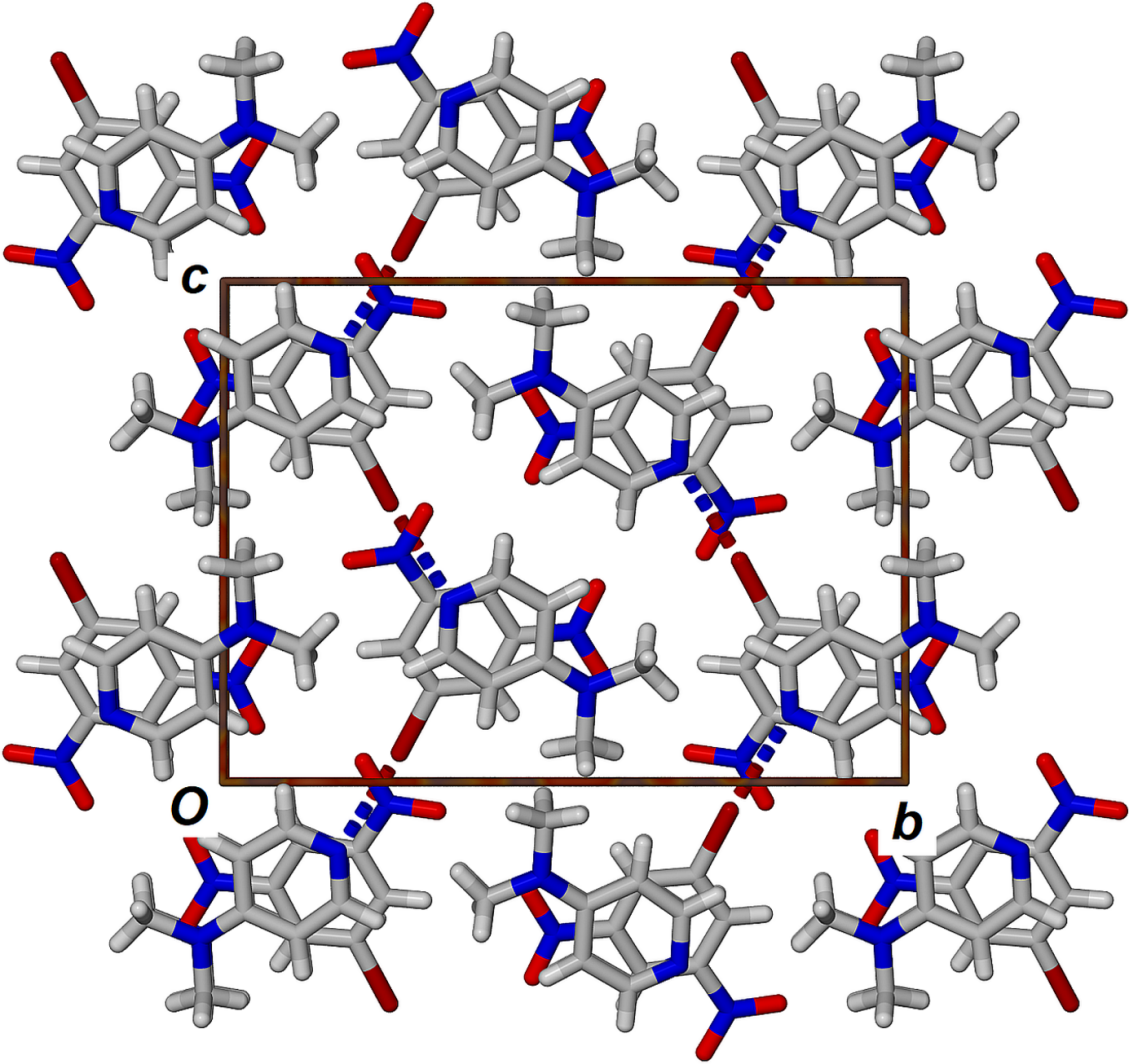
View of the crystal packing in the structure of **BDNB·DMAP** viewed along the *a* axis.

**Figure 3:**
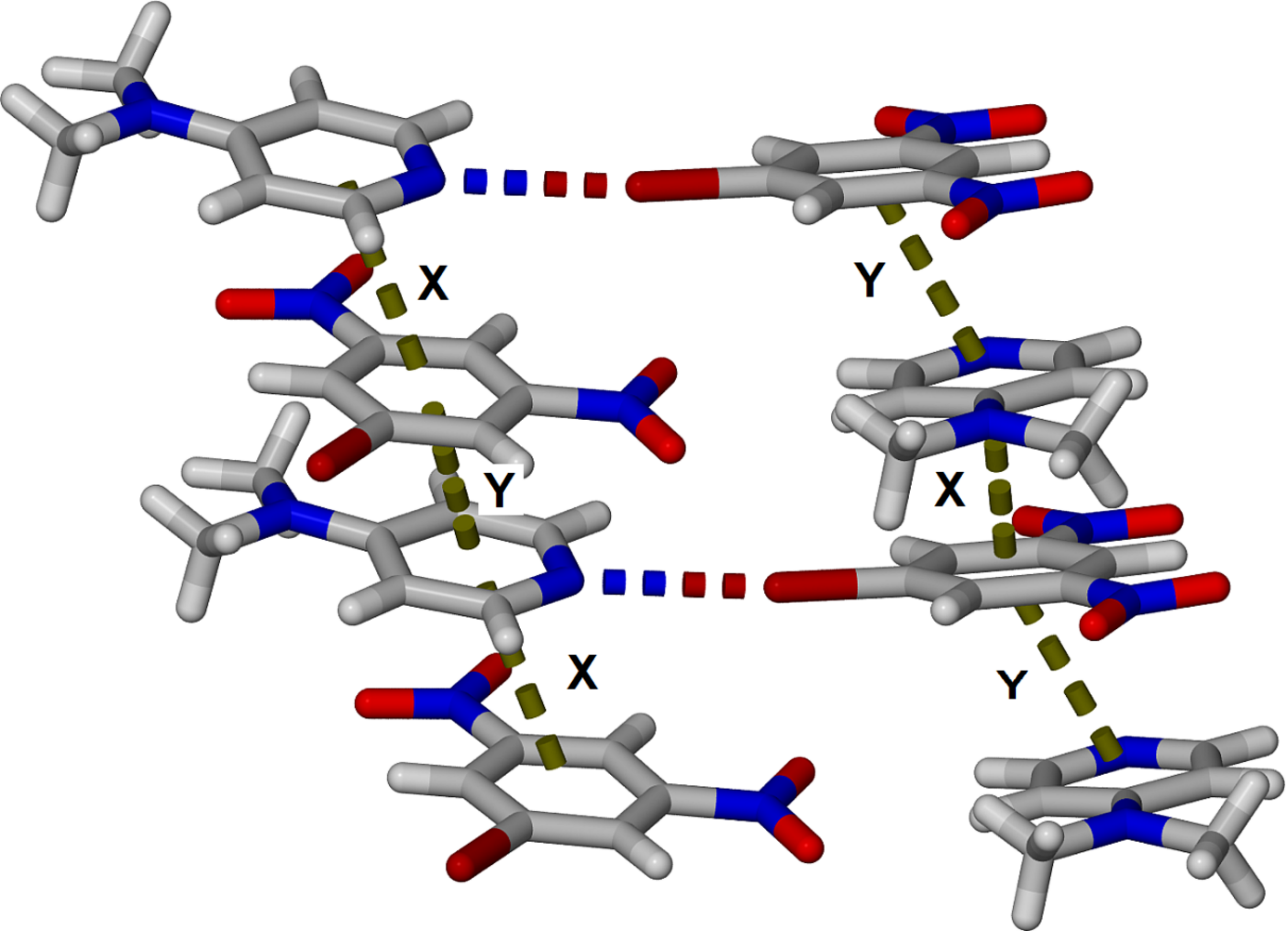
Oblique view showing the alternating π-stacking within the cocrystal **BDNB·DMAP** with two unique π–π inter­actions labeled x and y.

The unit-cell packing of **BDNB·DMAP** is shown in Fig. 2 ▸ highlighting the alternating π-stacking of the two components along the *a-*axis direction. The partial oblique view in Fig. 3 ▸ better illustrates the two unique π–π inter­actions. The inter­actions labelled ‘x’ and ‘y’ have centroid-to-centroid distances of 3.3639 (17) and 3.5926 (16) Å, respectively, with plane-to-plane centroid distances varying between 3.210 and 3.308 Å, confirming strong electron donor–acceptor π–π stacking of the aromatic rings.

**Figure 4:**
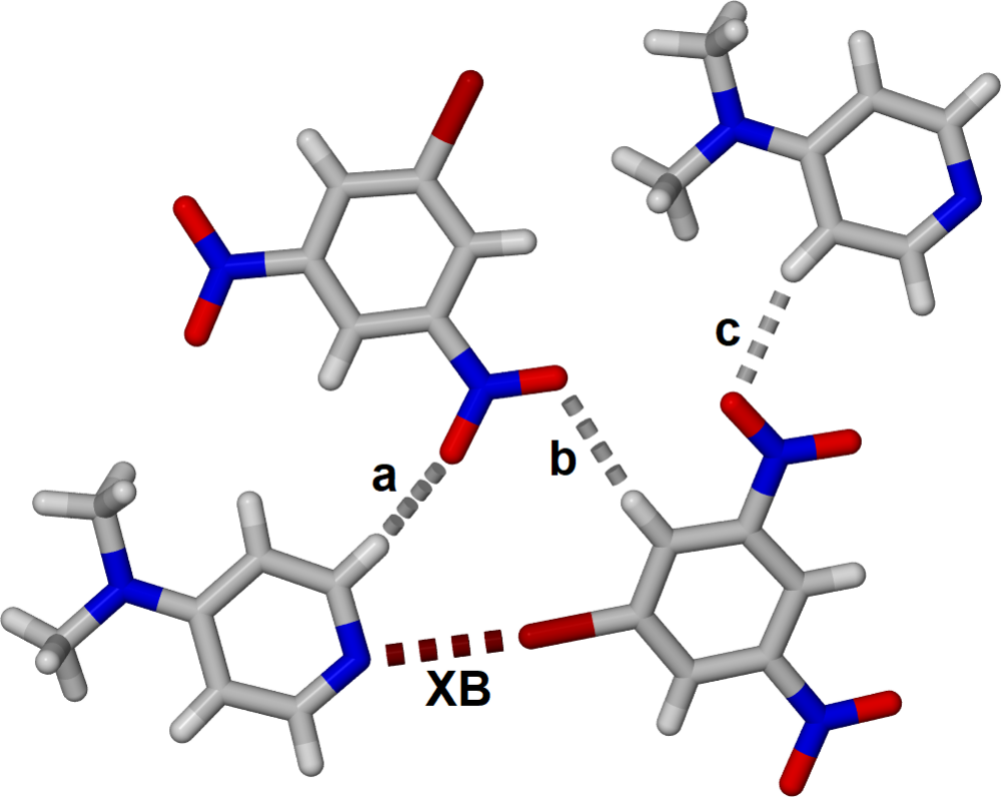
Inter­molecular inter­actions between adjacent mol­ecules in the structure of cocrystal **BDNB·DMAP**. C—H⋯O hydrogen bonds are labeled a, b and c, and the Br⋯N halogen bond is labeled XB

Within each plane there are secondary C—H⋯O hydrogen bonds (Table 1 ▸) as illustrated in Fig. 4 ▸. The H⋯O separations, a, b and c in Fig. 4 ▸, are C9—H9⋯O2^i^, C6—H6⋯O1^i^ and C7^ii^—H7^ii^⋯O4 are 2.46, 2.62 and 2.54 Å, respectively with C-H⋯O angles of 149, 171 and 160, respectively [symmetry codes: (i) −*x* + 1, *y* − 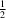, −*z* + 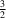; (ii) *x* − 1, *y*, *z* − 1]. These O⋯H separations correspond to 91, 96 and 93% of the sum of the van der Waals radii.

**Figure 5:**
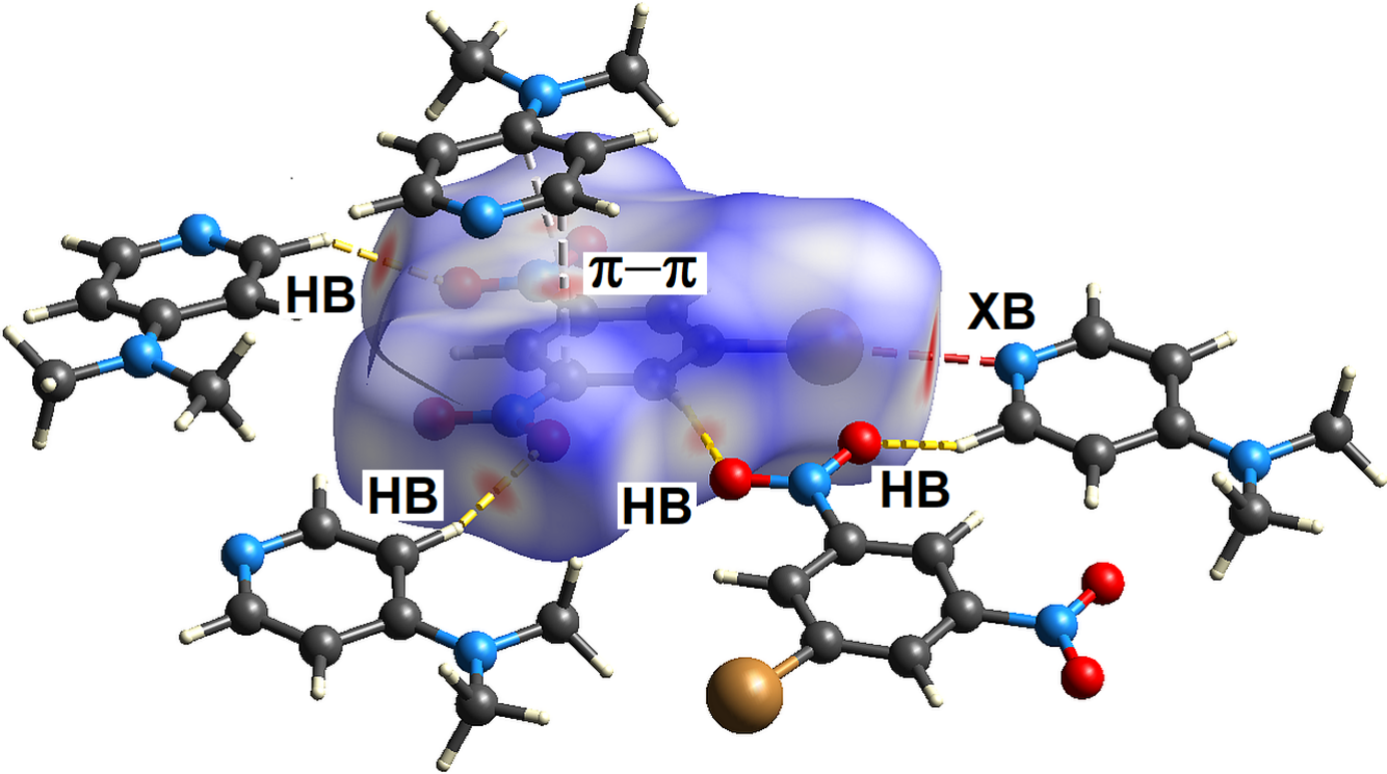
Hirshfeld surface for **BDNB** mapped over *d*_norm_. Red areas indicate close contacts with dashed lines showing atom-to-atom close contacts. Dashed lines are yellow for hydrogen bonding (HB), magenta for halogen bonding (XB) and gray for C⋯C π–π-stacking (π–π).

The program *CrystalExplorer21* (Spackman *et al.*, 2021 ▸) was used to calculate and plot the Hirshfeld surface of each mol­ecule within the cocrystal. The surface coloration is a visual representation of the inter­molecular atom-to-atom separation as compared to the sum of the van der Waals radii with close contacts colored red. Fig. 5 ▸ shows the Hirshfeld surface of the **BDNB** mol­ecule within the cocrystal. The adjacent mol­ecules responsible for close contacts to **BDNB** are correlated with hydrogen bonding, halogen bonding and π-stacking inter­actions as shown.

**Figure 6:**
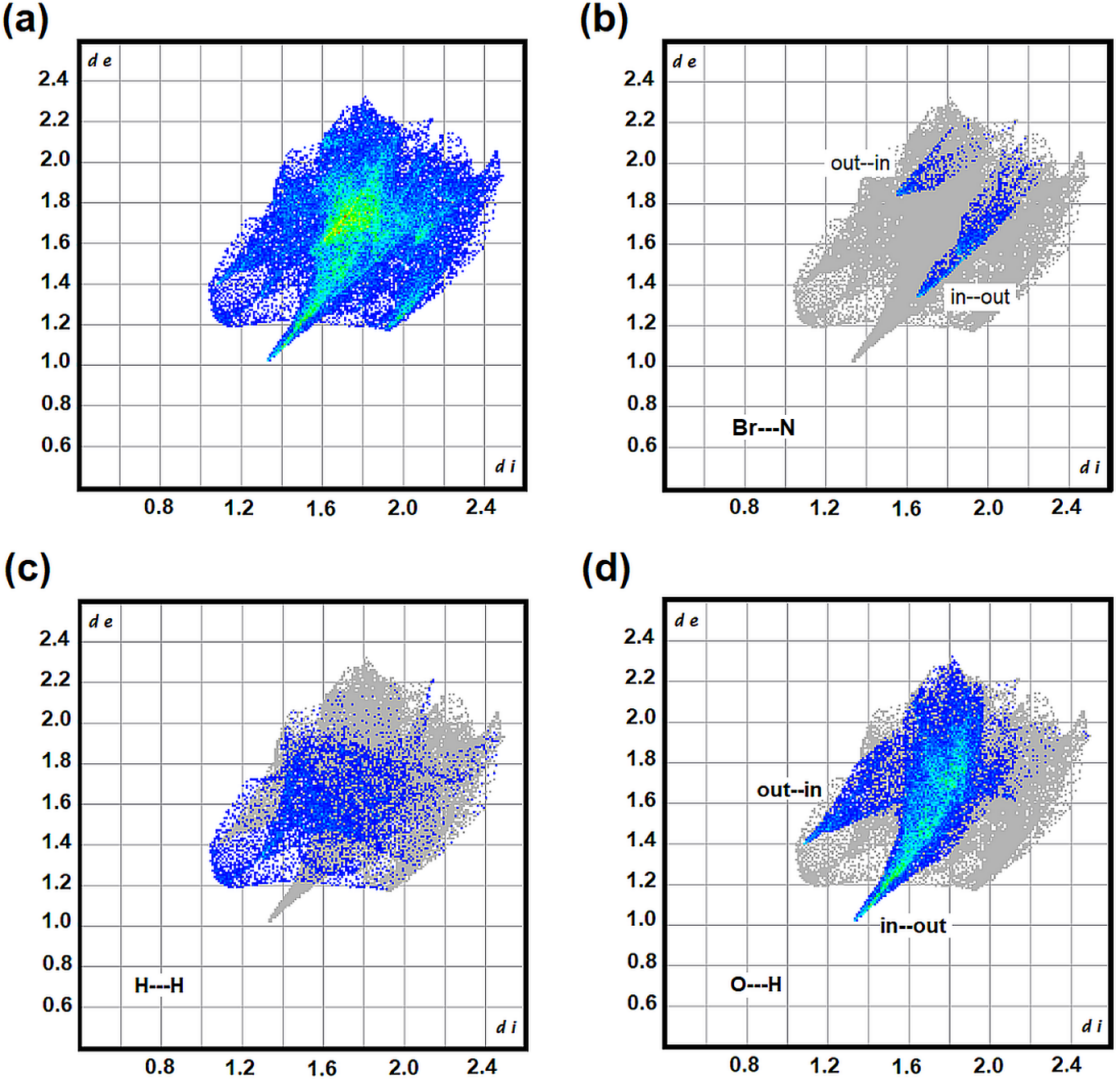
Two-dimensional fingerprint plots showing the contributions of the major inter­actions to the total Hirshfeld surface area of **BDNB** in cocrystal **BDNB·DMAP**. (*a*) All inter­actions, (*b*) Br⋯N, (*c*) H⋯H and (*d*) O⋯H In (*b*)–(*d*) reciprocal contacts are included and the in⋯out and out⋯in notation corresponds to the location of the first and the second atoms in order.

Fingerprint analysis was then used to breakdown the atom-to-atom inter­molecular contacts within the crystal as shown in Fig. 6 ▸ for the **BDNB** mol­ecule. The plot shown in Fig. 6 ▸(*a*) includes all atom-to-atom inter­actions and the light blue to green coloration corresponds to the most common inter­actions. Here these are concentrated in the range 3.2 to 3.6 Å typical for π-stacked aromatics. Breakdown of these contacts element-to-element reveals that the O⋯H inter­action along with the reciprocal H⋯O inter­action dominates, corresponding to 39.1% of the surface area, Fig. 6 ▸(*d*). Given that the **DMAP** mol­ecules have 10 H atoms on the periphery it is not surprising that H⋯H contacts are the second most common inter­action corresponding to 13.3% of the **BDNB** surface. Fig. 6 ▸(*c*) shows that some of these contacts are close to the sum of the van der Waals radii of 2.4 Å while others are significantly further up to 4.3 Å. The halogen bonding Br⋯N inter­action corresponds to only 3.4% of the surface area and the reciprocal inter­action, wherein an outer Br atom is in contact with an inner nitro N atom, increases this to 4.5% of the total surface area. Other significant inter­actions, and the percent of the surface area involved, include C⋯H (8.1%), C⋯C (5.6%) and O⋯Br (5.6%).

**Figure 7:**
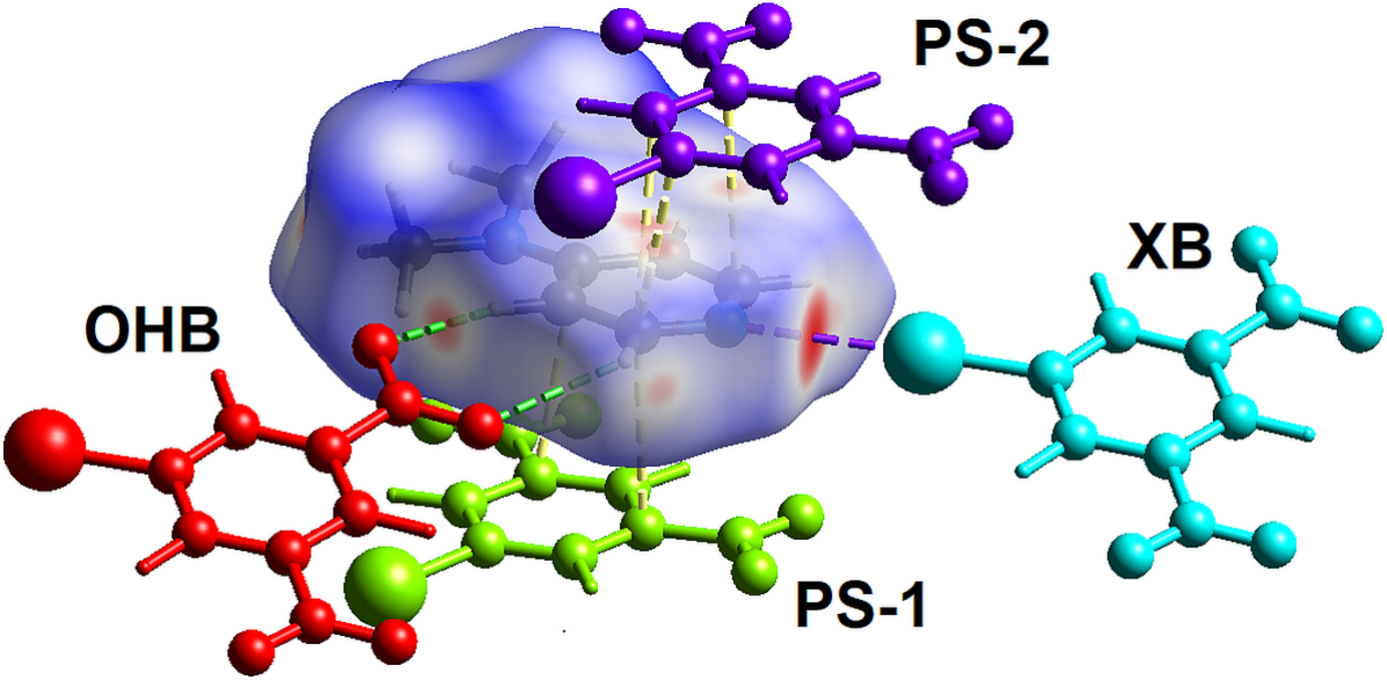
Color-coded mol­ecules, within 3.8 Å of a central **DMAP** mol­ecule in cocrystal **BDNB·DMAP**, that have significant attractive inter­molecular energies of inter­action. Mol­ecule color and labels are: PS-1 lime green, PS-2 purple, XB turquoise, and OHB red.

The program *CrystalExplorer21* was also used to calculate the inter­molecular inter­action energies between mol­ecules within the crystal structure. The total energy of inter­action between mol­ecules is expressed as the sum of four components: electrostatic, polarization, dispersion and exchange-repulsion. For this analysis, the mol­ecules within 3.8 Å of the **DMAP** mol­ecule in the asymmetric unit of cocrystal **BDNB·DMAP** were identified and the inter­molecular energy of inter­action between these mol­ecules and the central **DMAP** mol­ecule calculated. The four mol­ecules with the highest energy of inter­action with the **DMAP** mol­ecule are shown in Fig. 7 ▸. The two π-stacked mol­ecules, labelled PS-1 (lime green) and PS-2 (purple), have the strongest inter­molecular inter­action with total inter­action energies *E*_total_ of −45.8 and −42.3 kJ mol^−1^, respectively. The major cohesive components of the total energy for these mol­ecules are dispersion and electrostatic. The halogen bonded mol­ecule, turquoise and XB in Fig. 7 ▸, has an inter­mediate *E*_total_ of −17.7 kJ mol^−1^ with the major component being electrostatic while the dispersion is significantly less attractive given the small surface of this contact. The **BDNB** mol­ecule labelled OHB and red in Fig. 7 ▸, has *E_total_* = −9.2 kJ mol^−1^. This mol­ecule is C—H⋯O hydrogen bonded to the **DMAP** pyridine moiety with a similar dispersion component as the halogen-bonded mol­ecule but a greatly reduced electrostatic component.

These inter­molecular energies of inter­action are similar to those calculated in the same way for the previously reported analogous cocrystal **IDNB·DMAP** (Nwachukwu *et al.*, 2018 ▸). The iodo analogue also features two unique π-stacked inter­actions with slightly lower *E*_total_ values of −43.1 and −41.8 kJ mol^−1^, respectively, and, as expected, a stronger halogen-bonded mol­ecule with an *E_total_* of −27.5 kJ mol^−1^. These sytems were studied as we expected cooperative halogen bonding and enhanced π-stacking that could lead to colored cocrystals (Nwachukwu *et al.*, 2018 ▸).

## 4. Database survey

**Figure 8:**
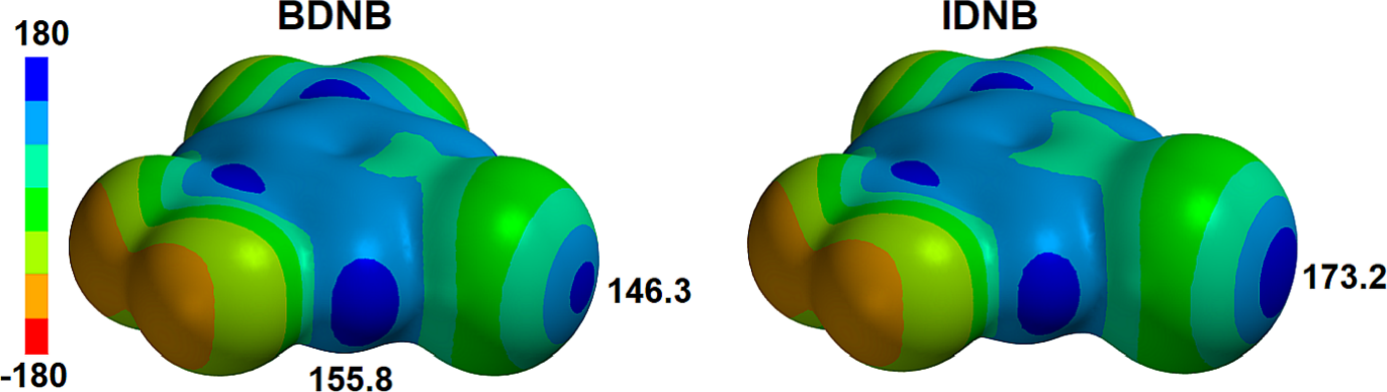
Mol­ecular electrostatic potential for **BDNB** and **IDNB** shown in kJ mol^−1^ calculated with *Spartan ’20* (Wavefunction, 2020 ▸) using density functional theory at the B3LYP-D3/6–311+G** level.

A search of the Cambridge Structural Database (ConQuest Version 2025.1.1, build 445489; Groom *et al.*, 2016 ▸) for inter­molecular C—*X*⋯N inter­actions where *X* is any halogen on a 1-halo-3,5-di­nitro­benzene and N a pyridyl N atom with unspecified substitution and *X*⋯N separation equal to, or less than, the sum of the van der Waals radii yielded six unique structures. All of these correspond to the halogen-bond donor 1,3-di­nitro-5-iodo­benzene (**IDNB**). These include the 1:1 cocrystal with 4,4-bipyridyl (Raatikainen & Rissanen, 2009 ▸) that features a halogen bond and a C—H⋯N hydrogen bond, two 1:1 cocrystals with 4-thio­phene-activated pyridines (refcodes: KETWOY and KETWUE; Nguyen *et al.*, 2018 ▸), the 1:1 cocrystal **IDNB·DMAP** we reported (refcode XIBNII; Nwachukwu *et al.*, 2018 ▸) and the 1:1 cocrystal with acridine (refcode: AXOKIK; Jain *et al.*, 2021 ▸). The last study also reports a ternary cocrystal of **IDNB** with **DMAP** and acridine (refcode: AXOHON) in which the **DMAP** forms a halogen bond while the acridine forms a C—H⋯N hydrogen bond to the H atom *para* to the iodine atom. The I⋯N separations in these examples range from 2.871 Å, for the halogen bond to the **DMAP** mol­ecule in the ternary cocrystal, to 3.072 Å in the cocrystal with the weaker base acridine as halogen-bond acceptor. Of particular relevance to this study, the I⋯N separation in cocrystal **IDNB·DMAP** is 2.892 Å, which is 82% of the sum of the van der Waals radii, lower than the 86% reported here for **BDNB·DMAP** indicative of a significantly stronger halogen bond. This is likely largely a reflection of the reduced σ-hole on the bromine atom as compared to the iodine atom (Fig. 8 ▸) in line with observations in other halogen-bond-donor systems.

## 5. Synthesis and crystallization

The compounds and solvents used in this study are available commercially and were used as received. Equimolar amounts, 0.1 mmol, of each component were weighed and placed in a small screw-cap vial and 2 mL of di­chloro­methane were added to effect complete solution of both compounds. The lid was loosely attached to permit slow evaporation of the solvent as a homogeneous mass of orange crystals formed.

## 6. Refinement

Crystal data, data collection and structure refinement details are summarized in Table 2 ▸. All H atoms were observed in the difference maps during refinement and added to C as riding atoms in geometrically idealized positions with C—H = 0.95 Å (aromatic) with *U*_iso_(H) = 1.2*U*_eq_(C) and 0.98 Å (meth­yl) with *U*_iso_(H) = 1.5U_eq_(C).

## Supplementary Material

Crystal structure: contains datablock(s) I. DOI: 10.1107/S2056989025006735/ev2020sup1.cif

Supporting information file. DOI: 10.1107/S2056989025006735/ev2020Isup3.cdx

Structure factors: contains datablock(s) I. DOI: 10.1107/S2056989025006735/ev2020Isup4.hkl

Supporting information file. DOI: 10.1107/S2056989025006735/ev2020Isup4.cml

CCDC reference: 2476608

Additional supporting information:  crystallographic information; 3D view; checkCIF report

## Figures and Tables

**Table 1:** Hydrogen-bond geometry (Å, °)

*D*—H⋯*A*	*D*—H	H⋯*A*	*D*⋯*A*	*D*—H⋯*A*
C6—H6⋯O1^i^	0.95	2.62	3.558 (3)	171
C7—H7⋯O4^ii^	0.95	2.54	3.444 (3)	160
C9—H9⋯O2^i^	0.95	2.46	3.314 (3)	149

Symmetry codes: (i) 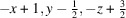; (ii) 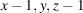.

**Table 2:** Experimental details

Crystal data	
Chemical formula	C_6_H_3_BrN_2_O_4_·C_7_H_10_N_2_
*M*_r_	369.18
Crystal system, space group	Monoclinic, *P*2_1_/*c*
Temperature (K)	100
*a*, *b*, *c* (Å)	7.0308 (8), 16.6382 (19), 12.4113 (14)
β (°)	99.665 (2)
*V* (Å^3^)	1431.3 (3)
*Z*	4
Radiation type	Mo *K*α
μ (mm^−1^)	2.90
Crystal size (mm)	0.20 × 0.02 × 0.02

Data collection	
Diffractometer	Bruker APEXI CCD
Absorption correction	Multi-scan (*SADABS*; Krause et al., 2015 ▸)
*T*_min_, *T*_max_	0.564, 0.746
No. of measured, independent and observed [*I* > 2σ(*I*)] reflections	17721, 3132, 2547
*R*_int_	0.056
(sin θ/λ)_max_ (Å^−1^)	0.641

Refinement	
*R*[*F*^2^ > 2σ(*F*^2^)], *wR*(*F*^2^), *S*	0.036, 0.092, 1.04
No. of reflections	3132
No. of parameters	201
H-atom treatment	H-atom parameters constrained
Δρ_max_, Δρ_min_ (e Å^−3^)	1.22, −0.55

Computer programs: *SMART* and *SAINT* (Bruker, 2014 ▸), *SHELXT2018/2* (Sheldrick, 2015*a* ▸), *SHELXL2019/2* (Sheldrick, 2015*b* ▸) and *X-SEED-4* (Barbour, 2020 ▸).

## supplementary crystallographic information

### 1-Bromo-3,5-dinitrobenzene–N,N-dimethylpyridin-4-amine (1/1) Crystal data

**Table:**

C_6_H_3_BrN_2_O_4_·C_7_H_10_N_2_	*F*(000) = 744
*M**_r_* = 369.18	*D*_x_ = 1.713 Mg m^−^^3^
Monoclinic, *P*2_1_/*c*	Mo *K*α radiation, λ = 0.71073 Å
*a* = 7.0308 (8) Å	Cell parameters from 3676 reflections
*b* = 16.6382 (19) Å	θ = 2.5–26.0°
*c* = 12.4113 (14) Å	µ = 2.90 mm^−^^1^
β = 99.665 (2)°	*T* = 100 K
*V* = 1431.3 (3) Å^3^	Irregular, yellow
*Z* = 4	0.20 × 0.02 × 0.02 mm

### 1-Bromo-3,5-dinitrobenzene–N,N-dimethylpyridin-4-amine (1/1) Data collection

**Table:**

Bruker APEXI CCD diffractometer	2547 reflections with *I* > 2σ(*I*)
Detector resolution: 8.3660 pixels mm^-1^	*R*_int_ = 0.056
φ and ω scans	θ_max_ = 27.1°, θ_min_ = 2.1°
Absorption correction: multi-scan (SADABS; Krause et al., 2015)	*h* = −8→9
*T*_min_ = 0.564, *T*_max_ = 0.746	*k* = −21→21
17721 measured reflections	*l* = −15→15
3132 independent reflections	

### 1-Bromo-3,5-dinitrobenzene–N,N-dimethylpyridin-4-amine (1/1) Refinement

**Table:**

Refinement on *F*^2^	Primary atom site location: dual
Least-squares matrix: full	Hydrogen site location: inferred from neighbouring sites
*R*[*F*^2^ > 2σ(*F*^2^)] = 0.036	H-atom parameters constrained
*wR*(*F*^2^) = 0.092	*w* = 1/[σ^2^(*F*_o_^2^) + (0.0543*P*)^2^] where *P* = (*F*_o_^2^ + 2*F*_c_^2^)/3
*S* = 1.04	(Δ/σ)_max_ = 0.001
3132 reflections	Δρ_max_ = 1.22 e Å^−^^3^
201 parameters	Δρ_min_ = −0.55 e Å^−^^3^
0 restraints	

### 1-Bromo-3,5-dinitrobenzene–N,N-dimethylpyridin-4-amine (1/1) Special details

**Table:**

Geometry. All esds (except the esd in the dihedral angle between two l.s. planes) are estimated using the full covariance matrix. The cell esds are taken into account individually in the estimation of esds in distances, angles and torsion angles; correlations between esds in cell parameters are only used when they are defined by crystal symmetry. An approximate (isotropic) treatment of cell esds is used for estimating esds involving l.s. planes.

### 1-Bromo-3,5-dinitrobenzene–N,N-dimethylpyridin-4-amine (1/1) Fractional atomic coordinates and isotropic or equivalent isotropic displacement parameters (Å^2^)

**Table:**

	*x*	*y*	*z*	*U*_iso_*/*U*_eq_	
Br1	0.31687 (4)	0.75730 (2)	0.55714 (2)	0.01681 (11)	
O1	0.3932 (3)	1.05122 (11)	0.72079 (17)	0.0251 (5)	
N1	0.4765 (3)	1.01510 (13)	0.80118 (19)	0.0187 (5)	
C1	0.4418 (4)	0.80802 (16)	0.6864 (2)	0.0146 (5)	
O2	0.5403 (3)	1.04681 (12)	0.88887 (17)	0.0273 (5)	
N2	0.7373 (4)	0.75831 (13)	0.95599 (19)	0.0189 (5)	
C2	0.4190 (4)	0.89029 (15)	0.6955 (2)	0.0158 (6)	
H2	0.348194	0.920532	0.637240	0.019*	
O3	0.8011 (3)	0.79467 (13)	1.04068 (16)	0.0251 (5)	
N3	0.1014 (3)	0.66874 (13)	0.36346 (18)	0.0181 (5)	
C3	0.5024 (4)	0.92726 (15)	0.7920 (2)	0.0160 (6)	
O4	0.7592 (3)	0.68655 (13)	0.94276 (17)	0.0313 (5)	
N4	−0.1420 (4)	0.46573 (13)	0.18916 (19)	0.0229 (6)	
C4	0.6039 (4)	0.88624 (16)	0.8795 (2)	0.0169 (6)	
H4	0.656153	0.912401	0.945946	0.020*	
C5	0.6250 (4)	0.80447 (16)	0.8647 (2)	0.0157 (6)	
C6	0.5464 (4)	0.76360 (15)	0.7707 (2)	0.0163 (6)	
H6	0.563356	0.707268	0.764146	0.020*	
C7	−0.0647 (4)	0.60854 (15)	0.1970 (2)	0.0159 (6)	
H7	−0.122556	0.616190	0.122847	0.019*	
C8	0.0127 (4)	0.67251 (16)	0.2590 (2)	0.0177 (6)	
H8	0.002635	0.723897	0.225159	0.021*	
C9	0.1113 (4)	0.59457 (16)	0.4066 (2)	0.0184 (6)	
H9	0.173796	0.588900	0.480293	0.022*	
C10	0.0381 (4)	0.52591 (16)	0.3526 (2)	0.0174 (6)	
H10	0.052278	0.475284	0.388440	0.021*	
C11	−0.0578 (4)	0.53166 (16)	0.2441 (2)	0.0166 (6)	
C12	−0.1001 (4)	0.38532 (16)	0.2326 (2)	0.0248 (7)	
H12A	0.039973	0.377412	0.248658	0.037*	
H12B	−0.157530	0.345532	0.178547	0.037*	
H12C	−0.154332	0.378825	0.299808	0.037*	
C13	−0.2364 (5)	0.47128 (18)	0.0760 (2)	0.0257 (7)	
H13A	−0.306021	0.522407	0.064445	0.039*	
H13B	−0.327598	0.426671	0.059330	0.039*	
H13C	−0.139295	0.468500	0.028008	0.039*	

### 1-Bromo-3,5-dinitrobenzene–N,N-dimethylpyridin-4-amine (1/1) Atomic displacement parameters (Å^2^)

**Table:**

	*U*^11^	*U*^22^	*U*^33^	*U*^12^	*U*^13^	*U*^23^
Br1	0.02091 (17)	0.01506 (15)	0.01351 (16)	−0.00126 (10)	0.00015 (11)	−0.00199 (10)
O1	0.0333 (12)	0.0139 (9)	0.0260 (11)	0.0024 (9)	−0.0015 (9)	0.0018 (8)
N1	0.0212 (13)	0.0133 (11)	0.0219 (13)	−0.0021 (9)	0.0038 (10)	−0.0006 (10)
C1	0.0148 (14)	0.0157 (12)	0.0132 (13)	−0.0032 (10)	0.0019 (11)	−0.0025 (10)
O2	0.0380 (13)	0.0194 (10)	0.0225 (11)	−0.0058 (9)	−0.0007 (9)	−0.0082 (9)
N2	0.0189 (12)	0.0231 (13)	0.0139 (12)	0.0025 (9)	0.0006 (9)	0.0021 (9)
C2	0.0168 (14)	0.0151 (13)	0.0153 (14)	0.0001 (10)	0.0020 (11)	0.0027 (10)
O3	0.0270 (12)	0.0296 (12)	0.0168 (11)	0.0000 (9)	−0.0022 (9)	0.0016 (9)
N3	0.0200 (12)	0.0174 (11)	0.0171 (12)	−0.0020 (9)	0.0037 (10)	−0.0048 (9)
C3	0.0196 (14)	0.0133 (12)	0.0154 (14)	−0.0007 (11)	0.0034 (11)	−0.0023 (10)
O4	0.0438 (14)	0.0223 (11)	0.0245 (12)	0.0082 (10)	−0.0034 (10)	0.0069 (9)
N4	0.0357 (15)	0.0138 (11)	0.0163 (13)	−0.0019 (10)	−0.0044 (11)	−0.0006 (9)
C4	0.0176 (14)	0.0204 (13)	0.0127 (13)	−0.0019 (11)	0.0029 (11)	−0.0020 (11)
C5	0.0156 (14)	0.0178 (13)	0.0138 (14)	0.0029 (11)	0.0026 (11)	0.0060 (10)
C6	0.0157 (14)	0.0150 (13)	0.0180 (14)	−0.0020 (10)	0.0029 (11)	0.0021 (11)
C7	0.0172 (14)	0.0175 (13)	0.0126 (14)	0.0002 (10)	0.0015 (11)	0.0016 (10)
C8	0.0189 (14)	0.0145 (13)	0.0206 (15)	0.0015 (11)	0.0065 (12)	0.0034 (11)
C9	0.0168 (14)	0.0237 (14)	0.0142 (14)	0.0018 (11)	0.0009 (11)	−0.0001 (11)
C10	0.0181 (14)	0.0166 (13)	0.0177 (15)	0.0000 (11)	0.0035 (11)	0.0029 (11)
C11	0.0161 (14)	0.0168 (13)	0.0166 (15)	−0.0015 (11)	0.0015 (11)	0.0006 (10)
C12	0.0338 (17)	0.0123 (13)	0.0276 (17)	−0.0002 (12)	0.0029 (14)	−0.0015 (11)
C13	0.0351 (18)	0.0243 (16)	0.0157 (15)	−0.0059 (13)	−0.0015 (13)	−0.0049 (12)

### 1-Bromo-3,5-dinitrobenzene–N,N-dimethylpyridin-4-amine (1/1) Geometric parameters (Å, º)

**Table:**

Br1—C1	1.893 (3)	C4—H4	0.9500
O1—N1	1.226 (3)	C5—C6	1.383 (4)
N1—O2	1.225 (3)	C6—H6	0.9500
N1—C3	1.480 (3)	C7—C8	1.371 (4)
C1—C2	1.385 (4)	C7—C11	1.404 (4)
C1—C6	1.387 (4)	C7—H7	0.9500
N2—O4	1.218 (3)	C8—H8	0.9500
N2—O3	1.230 (3)	C9—C10	1.380 (4)
N2—C5	1.482 (3)	C9—H9	0.9500
C2—C3	1.386 (4)	C10—C11	1.404 (4)
C2—H2	0.9500	C10—H10	0.9500
N3—C8	1.342 (3)	C12—H12A	0.9800
N3—C9	1.343 (3)	C12—H12B	0.9800
C3—C4	1.376 (4)	C12—H12C	0.9800
N4—C11	1.372 (3)	C13—H13A	0.9800
N4—C13	1.452 (3)	C13—H13B	0.9800
N4—C12	1.454 (3)	C13—H13C	0.9800
C4—C5	1.384 (4)		
			
O2—N1—O1	124.4 (2)	C8—C7—C11	119.4 (2)
O2—N1—C3	117.5 (2)	C8—C7—H7	120.3
O1—N1—C3	118.0 (2)	C11—C7—H7	120.3
C2—C1—C6	121.3 (2)	N3—C8—C7	125.6 (2)
C2—C1—Br1	117.8 (2)	N3—C8—H8	117.2
C6—C1—Br1	120.9 (2)	C7—C8—H8	117.2
O4—N2—O3	124.1 (2)	N3—C9—C10	125.2 (3)
O4—N2—C5	117.9 (2)	N3—C9—H9	117.4
O3—N2—C5	118.0 (2)	C10—C9—H9	117.4
C1—C2—C3	118.1 (2)	C9—C10—C11	119.3 (3)
C1—C2—H2	120.9	C9—C10—H10	120.3
C3—C2—H2	120.9	C11—C10—H10	120.3
C8—N3—C9	114.4 (2)	N4—C11—C7	122.7 (2)
C4—C3—C2	123.4 (2)	N4—C11—C10	121.3 (2)
C4—C3—N1	118.9 (2)	C7—C11—C10	116.0 (2)
C2—C3—N1	117.7 (2)	N4—C12—H12A	109.5
C11—N4—C13	121.2 (2)	N4—C12—H12B	109.5
C11—N4—C12	120.5 (2)	H12A—C12—H12B	109.5
C13—N4—C12	116.6 (2)	N4—C12—H12C	109.5
C3—C4—C5	115.8 (2)	H12A—C12—H12C	109.5
C3—C4—H4	122.1	H12B—C12—H12C	109.5
C5—C4—H4	122.1	N4—C13—H13A	109.5
C6—C5—C4	124.0 (2)	N4—C13—H13B	109.5
C6—C5—N2	118.4 (2)	H13A—C13—H13B	109.5
C4—C5—N2	117.6 (2)	N4—C13—H13C	109.5
C5—C6—C1	117.4 (2)	H13A—C13—H13C	109.5
C5—C6—H6	121.3	H13B—C13—H13C	109.5
C1—C6—H6	121.3		

### 1-Bromo-3,5-dinitrobenzene–N,N-dimethylpyridin-4-amine (1/1) Hydrogen-bond geometry (Å, º)

**Table:**

*D*—H···*A*	*D*—H	H···*A*	*D*···*A*	*D*—H···*A*
C6—H6···O1^i^	0.95	2.62	3.558 (3)	171
C7—H7···O4^ii^	0.95	2.54	3.444 (3)	160
C9—H9···O2^i^	0.95	2.46	3.314 (3)	149

Symmetry codes: (i) −*x*+1, *y*−1/2, −*z*+3/2; (ii) *x*−1, *y*, *z*−1.
